# Hybrid Welding (Laser–Electric Arc MAG) of High Yield Point Steel S960QL

**DOI:** 10.3390/ma14185447

**Published:** 2021-09-20

**Authors:** Michał Urbańczyk, Janusz Adamiec

**Affiliations:** 1 Łukasiewicz Resarch Network—Institute of Welding, Błogosławionego Czesława 16-18, 44-100 Gliwice, Poland; 2 Division of Engineering Materials, Faculty of Materials Engineering, Silesian University of Technology, Krasińskiego 8, 40-019 Katowice, Poland; Janusz.adamiec@polsl.pl

**Keywords:** hybrid welding, steel S960QL, HLAW, laser beam, MAG metal active gas

## Abstract

The article discusses the effect of the hybrid-welding process (laser–electric arc MAG *Metal Active Gas*) on the structure and properties of butt joints (having various thicknesses, i.e., 5 mm and 7 mm) made of steel S960QL. Welding tests were performed in the flat position (PA) and in the horizontal position (PC). Joints made of steel S960QL in the above-presented configuration are present in elements of crane structures (e.g., telescopic crane jibs). The welding tests involved the use of the G Mn4Ni1.5CrMo solid electrode wire and the Ar+18% CO_2_ shielding gas mixture (M21) (used in the MAG method). Non-destructive visual and radiographic tests did not reveal the presence of any welding imperfections in the joints. The welded joints obtained in the tests represented quality level B in accordance with the requirements of the ISO 12932 standard. Microscopic metallographic tests revealed that the heat-affected zone (HAZ) contained the coarse-grained martensitic structure resulting from the effect of the complex welding thermal cycle on the microstructure of the joints. Destructive tests revealed that the joints were characterised by tensile strength similar to that of the base material. The hybrid welding (laser–MAG) of steel S960QL enabled the obtainment of joints characterised by favourable plastic properties and impact energy exceeding 27 J. The tests revealed the possibility of making hybrid-welded joints satisfying the quality-related requirements specified in the ISO 15614-14 standard.

## 1. Introduction

**Figure 1:**
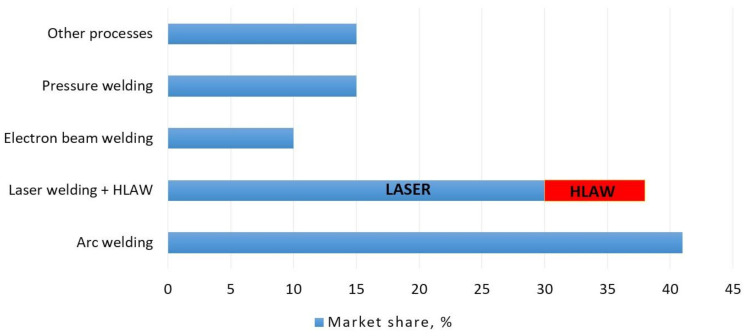
Various welding methods used in joining processes [6].

Presently, the implementation of advanced welding technologies in various industries is considered to be one of the most important trends enabling the modernisation of technological processes [1]. Laser welding is an advanced, continuously improved, and increasingly common welding process applied in numerous industries (Figure 1). The development of laser radiation sources has led to a situation where the market offer includes lasers having power exceeding 100 kW [2]. The laser-welding process and its variants (remote welding, hybrid welding, etc.) have become primary joining processes used in many industrial sectors [3,4,5].

**Figure 2:**
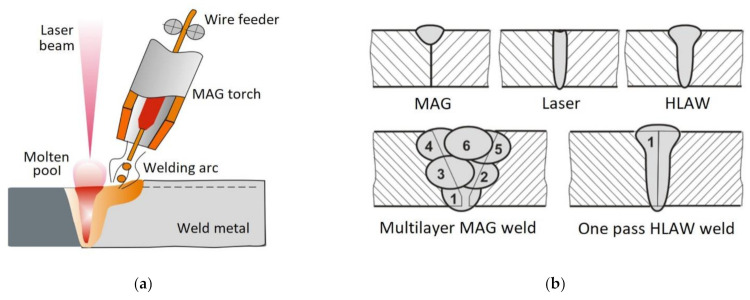
Hybrid laser arc welding (HLAW): (**a**) principle of the hybrid-welding process (laser–MAG *Metal Active Gas*) and (**b**) differences between the shape and geometry of welds obtained using the MAG welding, laser welding, and laser–MAG welding process [7].

One of the variants of the welding process using laser radiation as the heat source is hybrid laser arc welding (HLAW). The process involves the simultaneous use of two heat sources, i.e., laser radiation and electric arc. During the welding process, the use of the two above-named heat sources leads to the formation of the common weld pool (Figure 2a). The hybrid laser welding process involving the use of the combined heat source (laser beam and electric arc) is characterised by a number of advantages in comparison with the advantages characteristic of each of the aforementioned processes when used separately (Figure 2b) [7].

The first tests, aimed to combine two heat sources (i.e., laser radiation and electric arc), were first performed by Steen and Eboo in the 1970s [8,9]. The two scientists demonstrated that the simultaneous use of laser and electric arc enabled the obtainment of higher welding rates and greater penetration depth than those obtainable using laser welding and arc welding procedures separately. 

Obtainable results, the development of lasers, and the advantages resulting from the simultaneous use of two heat sources encouraged research centres worldwide to perform tests of hybrid technologies. 

Silva et al. (2020) [10] discussed the hybrid laser welding process performed using various values of laser radiation power. The authors demonstrated that the application of the hybrid-welding process enabled the obtainment of joints containing fewer welding imperfections (gas pores in the weld) in comparison with joints made using laser radiation only. In addition, hybrid-welded joints obtained by the authors were characterised by weld face hardness lower by 100 HV than that of the weld root, which, in turn, translated into reduced steel brittleness.

One of the crucial aspects of the hybrid-welding process is the manner of joint preparation. Kah et al. (2011) [11] described the effect of a gap located between elements being joined on the shape and geometry of the weld formed as a result of the hybrid-welding process. The authors demonstrated that an excessively large gap between the aforesaid elements (>0.8 mm) resulted in the reduced height of the weld face and the increased height of the weld root. 

Because of their advantages (significant penetration depth, higher welding rates, and reduced filler metal consumption), hybrid laser technologies are investigated by numerous research centres around the world. Researchers use HLAW to join various structural materials including steel [12,13], titanium alloys [14,15], aluminium alloys [16,17], or dissimilar materials [18,19,20].

Increased demand for high yield point structural steels (>900 MPa) in the crane-building industry has led to the intensification of tests concerning the applicability of hybrid-welding technologies in the joining of various steel grades [21,22]. 

Toughened steels having a yield point of 960 MPa are characterised by the fine-grained martensitic or martensitic-bainitic structure obtained through the toughening (i.e., hardening and quenching) of steel [23]. 

The joining of steels having the carbon equivalent value (CEV) ≤0.8% must be performed using processes characterised by a low heat input to the joint. According to Siltanen et al. (2011) [24], the hybrid-welding method (laser + electric arc) is rated among the aforesaid processes due to the reduction of beads needed to make a joint with full penetration (regarding the welding of elements having thickness >4 mm). The researchers conducted hybrid butt welding tests involving 6 mm thick plates made of steel S960QL. The use of the hybrid method resulted in lower heat input to the joint (only one bead was made) in comparison with that accompanying the use of the MAG method (where the obtainment of the same thickness required three beads). The mechanical properties of the joint were similar to those of the base material. 

The article discusses the effect of the hybrid-welding process (laser–electric arc MAG) on the structure and properties of butt joints having various thicknesses (i.e., 5 mm and 7 mm) and made of steel S960QL. Related welding tests were performed in the flat position (PA) and in the horizontal position (PC). Joints made of steel S960QL in the above-presented configuration are used in elements of crane structures (e.g., telescopic crane jibs) [25]. 

The hybrid-welding of plates having various thicknesses (e.g., 5 mm and 7 mm) made of high yield point steel S960QL and used in structural elements of cranes has been seldom discussed in scientific publications. Information on the subject is rudimentary and often subject to strict know-how-related confidentiality policy of individual manufacturers. The development of the crane industry and the growing demand for cranes have forced entrepreneurs to search for new high-performance welding technologies making it possible to increase production efficiency.

## 2. Materials and Methods

### 2.1. Materials

The tests discussed in the article involved the use of plates made of steel S960QL and having thicknesses of 5 mm and 7 mm; the remaining dimensions were 150 mm × 350 mm. The chemical composition of the plates was subjected to check analysis performed using a Q4 TASMAN 170 spark emission spectrometer (BRUKER; Billerica, MA, USA). The test results were compared with the requirements specified in the EN 10025-6+A1:2009 standard [26]. The results of the tests are presented in Table 1. 

The filler metal used in the welding of steel S960QL was a Union SG700 electrode wire (G Mn4Ni1.5CrMo: SG700/ID-No. 822000508, ISO 16834-A) having a diameter of 1.2 mm (Böhler Schweisstechnik). The shielding gas used in the MAG method was an M21 group gas mixture (Ar—82% and CO_2_—18%) (Messer). The shielding gas flow rate amounted to 16 dm^3^/min. 

### 2.2. Welding Method and Equipment

**Figure 3:**
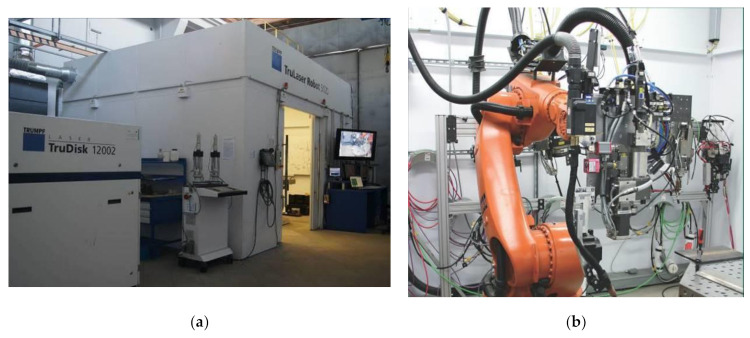
Robotic laser welding station (TruLaser Robot 5120) with the TruDisk 12002 disc laser: (**a**) main view, (**b**) D70 hybrid-welding head (Trumpf) (Łukasiewicz Research Network—Institute of Welding in Gliwice).

The hybrid-welding (laser–MAG) tests were performed at Łukasiewicz Research Network—Institute of Welding using a robotic welding station (Figure 3a) consisting of a TruDisk 12002 disc laser (TRUMPF; Stuttgart, Germany) having a power of 12 kW, a KRC30HA welding robot (KUKA; Augsburg, Germany) equipped with a hybrid-welding head (Figure 3b), and a PHOENIX 452 RC PULS MIG/MAG welding machine generating a maximum welding current of 450A (EWM Hightec Welding GmbH; Mündersbach, Germany).

**Figure 4:**
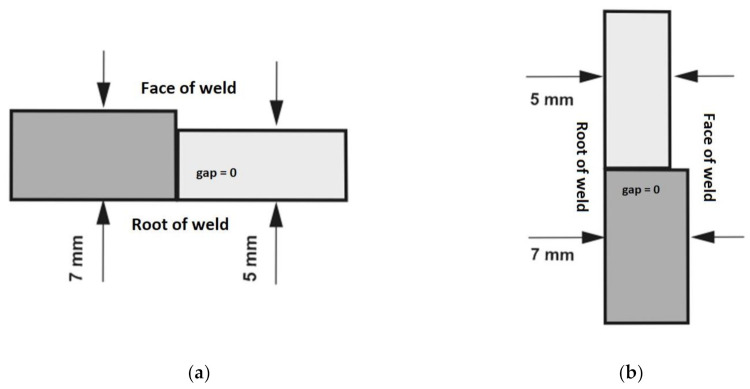
Setting up and plate thicknesses used in the tests: (**a**) flat position (PA) and (**b**) horizontal position (PC) (according to ISO standard positions).

The welding tests were performed in two positions, i.e., in the flat position (PA) (Figure 4a) and in the horizontal position (PC) (Figure 4b). The edges of the plates to be joined were subjected to square butt weld preparation and set up without a gap (b = 0) at the interface.

**Figure 5:**
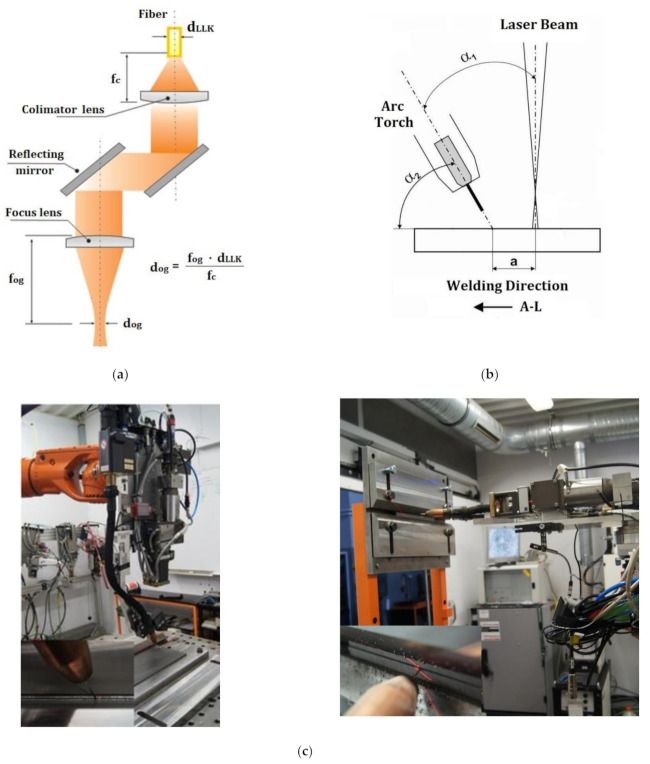
Schematic diagram of the laser optical system as well as the position of the laser beam and electric arc in the hybrid-welding technology: (**a**) laser optical system, (**b**) position of the laser beam and electric arc, and (**c**) processing head inclination during welding in the flat position (PA) and in the horizontal position (PC).

During the hybrid-welding process, the laser radiation beam was transported using an optical fibre (dLLK) having a diameter of 400 µm and enabling the obtainment of laser beam focus diameter d_og_ = 0.8 mm (in relation to f_col_ = 200 mm—collimator lens focal length and f_og_ = 400 mm—focusing lens focal length; Figure 5a).

The laser radiation beam was positioned in the plane perpendicular to the surface of the plates, whereas the MAG welding torch was positioned at an angle of 65° (α1) in relation to the surface of the plates. The angle between the beam axis and the axis of the MAG welding torch (α2) amounted to 25°. The distance between the laser beam focus and the electrode tip (a) amounted to 2 mm (Figure 5b). During welding performed in the horizontal position (PC), the entire system was inclined at an angle of 90° (Figure 5c).

The welding tests were performed in the A–L (arc leading) configuration, i.e., with the arc power source leading the heat source in the process (Figure 5b). 

A heat input was calculated using Equation (1) presented in the ISO 15614-14 standard [27].

(1) $$Q=\frac{\left(P_{\mathrm{laser}}\cdot U\cdot I\right)}{v_{s}}\cdot 10^{-3}\left[\mathrm{kJ}/\mathrm{mm}\right]$$

where *Q*—heat input (kJ/mm), P_laser_—laser power (W), U—arc voltage (V), I—welding current (A), and v_s_—welding rate (mm/s).

The setting up of the plates along with their thicknesses in the welding tests performed in various welding positions, are presented in Figure 4.

### 2.3. Tests of Welded Joints

The joints were subjected to visual tests (VT), radiographic tests (RT), and destructive tests performed in accordance with the requirements of the ISO 15614-14 standard (concerning hybrid-welding procedure qualification). The results of observations and measurements performed to identify the quality level (regarding the presence of welding imperfections) were assessed in accordance with the ISO 12932 standard [28]. 

The research included the following tests:

- Visual tests performed in accordance with the requirements of the ISO 17637 standard;
- Radiographic tests of the welded joints, performed in accordance with the requirements of the ISO 17636-1 standard and involving the use of an Eresco 65 MF3 X-ray unit (GE Sensing&Inspection Technologies; Ahrensburg, Germany);
- Macroscopic metallographic tests, performed using an Olympus SZX9 stereoscopic microscope (Olympus, Tokyo, Japan). To identify their structure, the specimens were subjected to etching in Adler’s reagent (Chmes, Poznań, Poland);
- Microscopic metallographic tests, performed in accordance with the requirements of the ISO 17639 standard and involving the use of a Nikon Eclipse MA200 light microscope (Leuven, Belgium). The specimens were subjected to grinding with abrasive paper having a granularity of 800 and 1000, polishing performed using a powerpro 4000 grinder/polisher (Buehler; Germany) and metaldi Monocrystalline Diamond Suspension (3 µm), as well as etching in 5% Nital (5% HNO3 in ethanol);
- Tests performed using a scanning transmission electron microscope (STEM) involving the use of thin foils; the specimens were subjected to two-sided grinding (with abrasive paper) to reach a thickness of 0.5 mm. The process of electrochemical thinning was performed using a Struers tenupol-5 machine, with a voltage of 45 V and a temperature of 5 °C. The process was carried out in electrolyte composed of 70% CH20H, 20% glycerine, and 10% hclo4. The cooling agent was liquid nitrogen. The tests were performed using a Hitachi 2300A scanning-transmission electron microscope (STEM) (Japan), illuminating thin foils. The microscope was equipped with an FEG-type gun provided with the Schottky emitter. The accelerating voltage during the tests amounted to 200 kv;
- Hardness distribution tests were performed in accordance with the requirements of the ISO 9015-1 standard and involved the use of a GNEHM DIGITAL BRICKERS 220 hardness tester. Vickers hardness tests (HV) were performed along two measurement lines located 2 mm away from the upper and lower edge of the specimen. The imprints were made in the base material, heat-affected zone (HAZ) and in the weld;
- Static tensile tests involved 2 specimens cut out perpendicularly to the weld and prepared in accordance with the requirements of the ISO 6892-1 standard. The preparation of the specimens involved the removal of excessive root and face reinforcement as well as the mechanical reduction of specimen thickness from 7 mm to 5 mm (performed to obtain the even surface of the plates across the entire specimen). The dimensions of the specimens were 300 mm × 25 mm × 5.0 mm. The tension rate amounted to 10 mm/min. The tests were performed using an MTS 810 TEST SYSTEMS testing machine (Eden Prairie, MN, USA);
- Face bend test (FBB) and root bend test (RBB) of the butt weld were performed in accordance with the requirements of the ISO 5173 standard. The tests involved 4 specimens—two specimens on each side. The thickness of the plate was mechanically reduced from 7 mm to 5 mm (in order to obtain the even surface of the plates across the entire specimen). The dimensions of the specimens were 300 mm × 20 mm × 5.0 mm). The tests were performed using a LOS12126 testing machine (Losenhausenwerk AG; Düsseldorf, Germany);
- Impact strength tests, performed in accordance with the requirements of the ISO 9016 standard, involved the use of 2 sets of specimens (3 specimens in each set) sampled from the weld area and from the heat-affected zone (HAZ). The cross-section of the specimens used in the test was reduced. The dimensions of the specimens were 2.5 mm × 8.0 mm × 55 mm. The depth of the V notch amounted to 2 mm. Before the tests, the specimens were cooled to a temperature of −40 °C. The cooling process was performed using an FP89 cooling circulator (Julabo). Impact energy was identified using an RKP 300 impact-testing machine (Amsler).

The parameters of the hybrid-welding process (laser + electric arc MAG) performed both in the flat position (PA; joint no. 1) and in the horizontal position (PC; joint no. 2) are presented in Table 2.

## 3. Results and Discussion

### 3.1. Weld Formation

**Figure 6:**
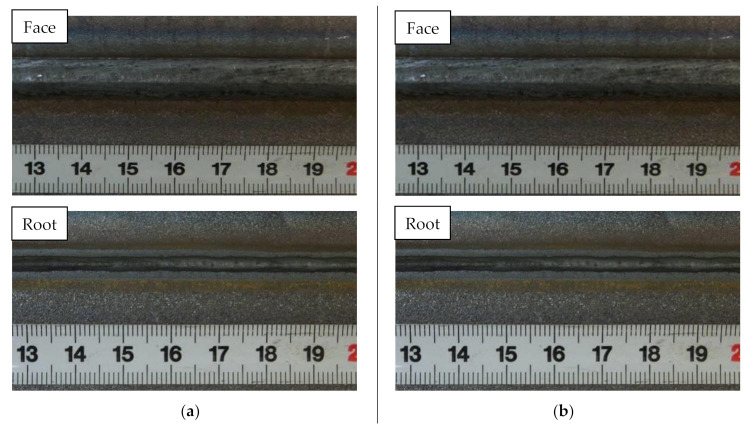
Joint (having thickness 5 mm and 7 mm) viewed from the face side and the root side after the hybrid-welding process: (**a**) joint no. 1 (PA) and (**b**) joint no. 2 (PC) (in accordance with Table 2).

The weld face side and the weld root side of the joints after the hybrid-welding process are presented in Figure 6. 

The visual welding tests revealed that hybrid-welded joints no. 1 and 2 (Figure 6) made in the flat position (PA) and in the horizontal position (PC) were characterised by the smooth spatter-free weld face and the properly shaped weld root. 

In accordance with the requirements specified in the ISO 12932 standard (concerning hybrid-welding procedure qualification), the joints made in the PA and PC positions satisfied related criteria and represented quality level B.

**Figure 7:**
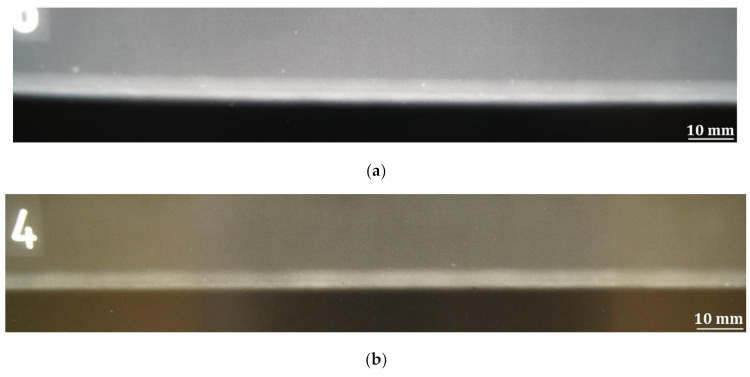
X-ray photograph of the hybrid-welded joints: (**a**) joint no. 1 (PA) and (**b**) joint no. 2 (PC).

The subsequent stage included the performance of non-destructive radiographic (X-ray) tests aimed to detect (if any) internal welding imperfections. The X-ray tests involved 100% of the joint length. The X-ray photographs of joints no. 1 and 2 are presented in Figure 7. Joint no. 1 and joint no. 2 did not contain any internal welding imperfections.

**Figure 8:**
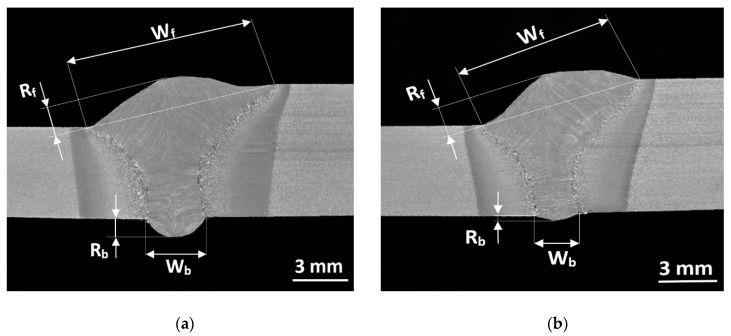
Macrostructure as well as the face and the root of the hybrid-welded joints (having thicknesses of 5 mm and 7 mm): (**a**) joint no. 1 (PA) and (**b**) joint no. 2 (PC).

The subsequent stage involved macrostructural tests of joints no. 1 and 2 (Figure 8). The macrostructural tests did not reveal any welding imperfections within the weld area and in the heat-affected zone (HAZ). 

The etched metallographic specimens revealed the clearly visible borders between the base material, HAZ, and the weld.

Geometrical dimensions of hybrid-welded joints no. 1 and 2 made in the flat position (PA) and in the horizontal position (PC) are presented in Table 3.

The width of the weld face (W_f_/mm) of the joint made in the flat position (PA) amounted to 9.7 mm, whereas its height (R_f_/mm) amounted to 1.5 mm. The width of the weld root (W_b_/mm) amounted to 3.1 mm, whereas its height (R_b_/mm) amounted to 1 mm. The width of the weld face (W_f_/mm) of the joint made in the horizontal position (PC) amounted to 8.3 mm and was 1.4 millimetres lower in comparison with that of the joint made in the flat position (PA). The height of the weld face (R_f_/mm) amounted to 1.7 mm. The width of the weld root (W_b_/mm) of the joint made in the horizontal position (PC) amounted to 2.2 mm (0.9 mm less than that of the joint made in the flat position (PA)). The height of the weld root (R_b_/mm) amounted to 0.3 mm (0.7 mm less than that of the joint made in the flat position (PA)) (Table 3).

The tests revealed that joints no. 1 and 2 represented quality level B in accordance with the requirements specified in the ISO 12932 standard (concerning hybrid-welding procedure qualification). 

### 3.2. Microstructure Characteristics

**Figure 9:**
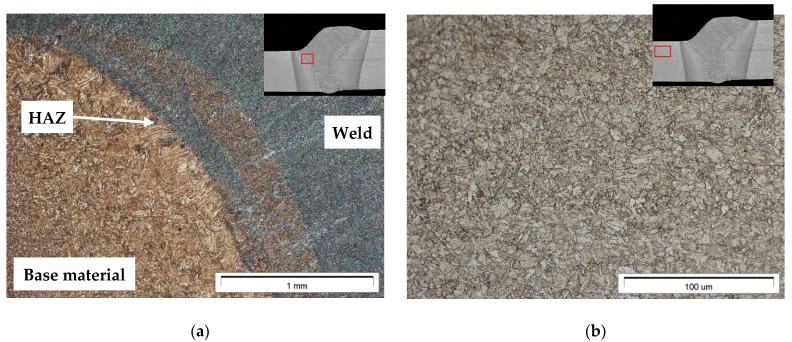

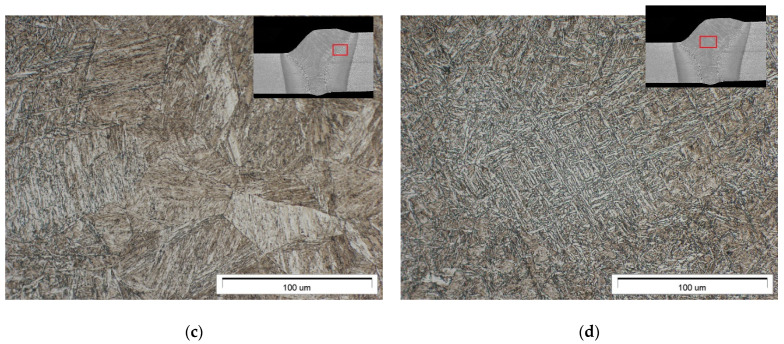
Structure of hybrid-welded joint no. 1 made of steel S960QL: (**a**) macrostructure, HAZ, and the weld; (**b**) fine-grained martensite in the base material; (**c**) HAZ containing coarse-grained martensit; (**d**) weld—martensitic structure.

Joint no. 2 (PC) was subjected to microscopic metallographic tests. The microscopic tests revealed the presence of three typical areas (Figure 9a), i.e., the base material (containing the fine-grained structure of tempered martensite (Figure 9b), the heat-affected zone having a width of approximately 1 mm (Figure 9c), and the weld area (Figure 9d).

**Figure 10:**
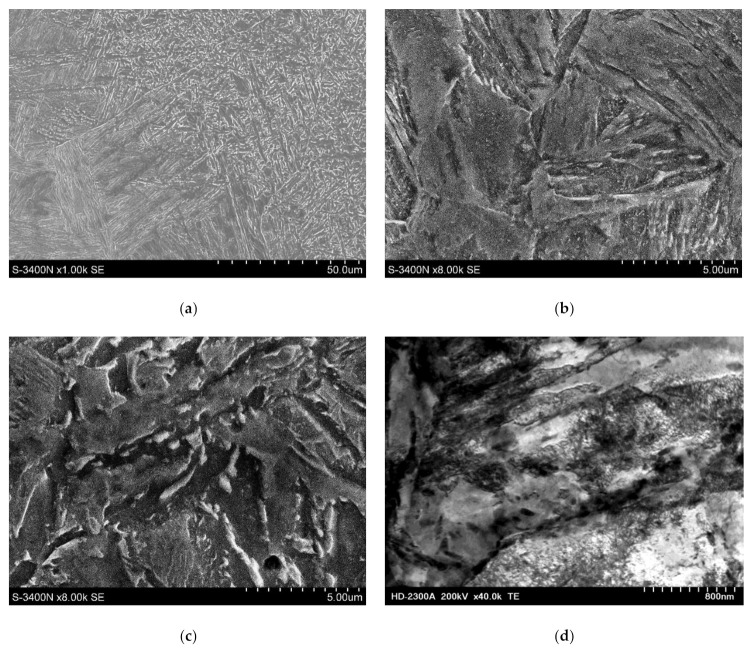

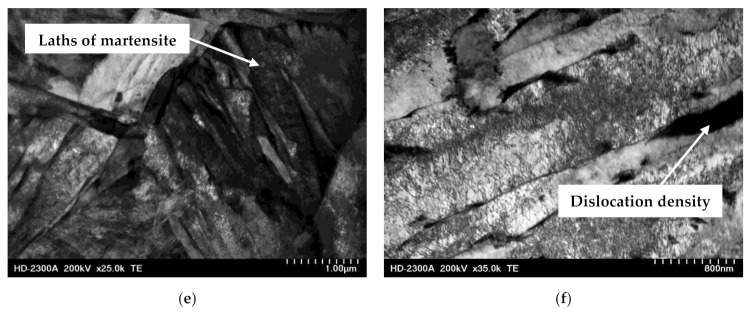
Microstructure of the welded joint made of steel S960QL: (**a**) fusion line (SEM), (**b**) HAZ (SEM), (**c**) weld (SEM), (**d**) base material—martensite with visible dislocations (STEM), (**e**) martensite observed in the HAZ (STEM), and (**f**) laths of martensite in the weld with a visible increase in dislocation density (STEM).

The heat-affected zone (HAZ) contained the coarse-grained martensitic structure (having a thickness of 425 HV10) formed as a result of the welding thermal cycle effect (Figure 9c). In turn, the weld contained the homogenous acicular martensitic structure having a hardness of 350 HV10 (Figure 8d). The above-presented observation results were confirmed by tests performed using a scanning transmission electron microscope (Figure 10a–f).

In article [29], Guo et al. stated that the crucial aspects related to the welding of fine-grained, high-strength steel S960 MPa were the temperature of the process and the welding rate. Those two factors significantly affected the microstructure in the zones of the welded joint (HAZ, weld). 

In the structure of the base material (in the martensitic laths), it was possible to observe significant dislocation density, which was connected with the manufacturing and hardening of high yield point steel S960QL. During welding, the thermal cycle effect triggered austenitisation and the growth of austenite grains in the HAZ. The cooling of the heat-affected zone led to the martensitic transformation, resulting in the formation of coarse-grained martensite (Figure 10b). The aforesaid area also contained fine carbides (Figure 10e). The weld contained fine-grained martensite with numerous fine carbides (Figure 10c). The martensite laths were characterised by significant dislocation density (indicating the hardening of the joint in the above-named area).

### 3.3. Hardness Distribution

Hardness measurements concerning joint no. 1 (PA) and joint no. 2 (PC) revealed that the highest hardness value was characteristic of the weld–HAZ interface. In relation to joint no. 1, the aforesaid hardness amounted to 436 HV10, whereas in relation to joint no. 2, the hardness amounted to 448 HV10. An increase in hardness in the aforesaid area could be ascribed to the rate of heat propagation and the hardening of the joint. A similar distribution of hardness in hybrid-welded joints made of fine-grained high-strength steel was observed by Lahdo et al. (2014) [30]. The authors stated that the weld–HAZ interface underwent hardening, which was connected with the rate at which the above-named area was cooled. The reduction of hardness at the weld–HAZ interface would require the application of preheating. 

**Figure 11:**
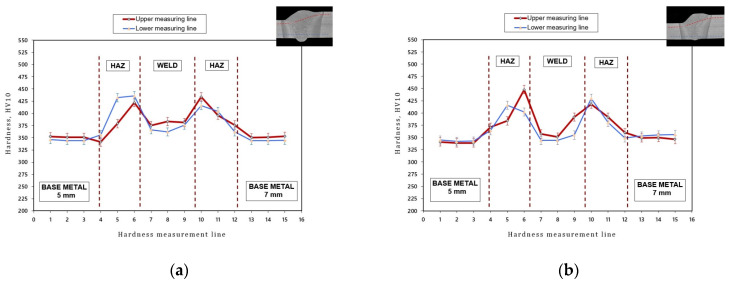
Measurement results and the distribution of hardness in the cross-section of the HLAW joint: (**a**) joint no. 1 (PA) and (**b**) joint no. 2 (PC).

The difference in hardness between the base material and the HAZ in the joints made in the flat position and in the horizontal position amounted to 22%. Regarding joint no. 2, hardness in the central area of the weld was similar to that of the base material and amounted to 344 HV10 (Figure 11b). In turn, regarding joint no. 1, the difference between the above-named zones amounted to 20 HV10 (Figure 11a). 

Both in the joint made in the flat position (PA) and that made in the horizontal position (PC), the HAZ was characterised by Vickers hardness above 400 (448 HV10). The above-presented values are acceptable in accordance with the requirements of the ISO 15614-14 standard concerning hybrid-welding procedure qualification. The hardness increase in the aforesaid area did not affect other mechanical properties (tension, bending).

### 3.4. Mechanical Properties

**Figure 12:**
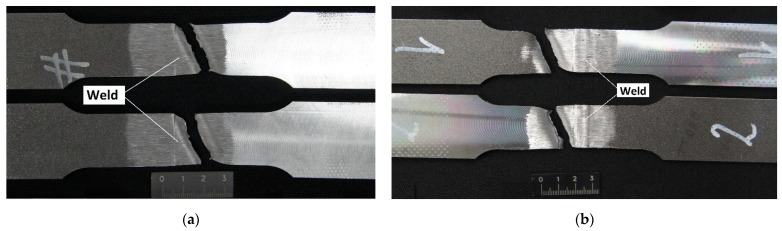
Specimens after the static tensile test of the HLAW joint: (**a**) joint no. 1 (PA) and (**b**) joint no. 2 (PC).

The analysis of the destructive tests concerning the hybrid-welded joints (laser beam—MAG) revealed that both joint no. 1 and joint no. 2 satisfied the requirements of the ISO 15614-14 standard (Table 4). The hybrid-welding process did not trigger a decrease in the tensile strength of the joints (1053 MPa and 1068 MPa, respectively) in comparison with that of the base material (restricted within the range of 980 MPa to 1150 MPa; Table 1). The specimens ruptured in the HAZ (area characterised by a clearly visible grain growth). The decrease in tensile strength in the HAZ area resulted from the loss of mechanical properties obtained by the steel in the manufacturing process (as a result of hardening and tempering). Figure 12 presents the specimens after the static tensile test of the HLAW joint.

The bend angle obtained in the bend test amounted to 180°—both during the face and root bend tests of the butt weld. The joints were characterised by high plastic properties. 

Impact energy obtained at a temperature of −40 °C also indicated the favourable mechanical properties of the joints. In relation to the specimens sampled from the central area of the weld, both regarding joint no. 1 (PA) and joint no. 2 (PC), impact energy amounted to 30 J. In turn, regarding the specimens sampled from the HAZ area, impact energy amounted to 46 J in relation to the joint made in the flat position and 40 J in relation to the joint made in the horizontal position.

Siltanen et al. (2015) [31] obtained similar mechanical properties using another filler metal (in the hybrid-welding process). In relation to 6 mm thick hybrid-welded joints, the tensile strength amounted to 1000 MPa, whereas impact energy exceeded 27 J (34 J, 48 J) (at a testing temperature of −40 °C). Therefore, the joints satisfied the requirements specified in the EN 10025-6 standard [26].

## 4. Conclusions

The hybrid-welding tests (laser + MAG) involved the making and testing of butt joints with full penetration. The joints (having various thicknesses, i.e., 5 mm and 7 mm) were made of steel S960QL plates. The above-named steel is used, among other things, in the production of telescopic jibs of self-propelled cranes.

The tests revealed the possibility of obtaining welded joints satisfying the requirements of quality level B (i.e., top quality level) in accordance with the ISO 15614-14 standard.

Both the joint made in the flat position (PA—1G) and that made in the horizontal position (PC—2G) were characterised by the uniform, smooth, and spatter-free weld face as well as by the properly shaped weld root. The visual and radiographic tests did not reveal the presence of surface or internal welding imperfections.

The static tensile tests revealed that the joints were characterised by high strength (1053 MPa and 1068 MPa, respectively) and high plasticity (bend angle of 180°).

The specimens sampled from the weld area and from the heat-affected zone revealed that impact energy amounted to more than 27 J (30 J in relation to the weld as well as 40 J and 46 J in relation to the HAZ).

The microscopic metallographic tests revealed that the heat-affected zone (HAZ) contained the coarse-grained martensitic structure formed as a result of the complex welding thermal cycle effect. During welding, the thermal cycle effect triggered austenitisation, the growth of austenite grains and the precipitation of carbides in the heat-affected zone. In turn, the weld contained the homogenous structure of acicular martensite.

## Figures and Tables

**Table 1:** Chemical composition and mechanical properties of the test plates made of steel S960QL.

Chemical Composition, (%)											
	C	Si	Mn	P	S	Cr	Cu	Ni	Mo	V	CEV
EN 10025-6	max 0.20	max 0.80	max 1.70	max 0.02	max 0.01	max 1.5	max 0.50	max 2.0	max 0.70	-	max 0.82
Check analysis	0.13	0.39	1.40	0.009	0.001	0.01	0.01	0.19	0.44	0.03	0.47
Mechanical properties											
R_m_ [MPa]				R_e_ [MPa]				A_5_ [%]			
980 ÷ 1150				960				10			

**Table 2:** Parameters of the hybrid-welding process used when making the joints (having thicknesses of 5 mm and 7 mm) in the flat position (joint no. 1 PA) and horizontal position (joint no. 2 PC).

Welding Parameters	Joint No. 1 (PA)	Joint No. 2 (PC)
Laser power (kW)	3.75	3.75
Welding rate (m/min)	1.3	1.3
Filler metal wire feed rate (m/min)	8.5	8.5
Welding current (A)	290	275
Arc voltage (U)	27	27
Interface gap (between the plates) (mm)	0	0
Heat input (kJ/mm)	0.57	0.56

**Table 3:** Geometrical dimensions of hybrid-welded joints no. 1 and 2.

Dimensions	Joint No. 1	Joint No. 2
Weld face width (W_f_/mm)	9.7	8.3
Weld face height (R_f_/mm)	1.5	1.7
Weld root width (W_b_/mm)	3.1	2.2
Weld root height (R_b_/mm)	1	0.3

**Table 4:** Mechanical test results concerning the hybrid-welded joints made of steel S960QL.

Joint No.	Tensile Strength *, ^1^		Bend Test *, Bend Angle, °		Impact Strength Test KCV **, Impact Energy J, (Testing Temperature: −40 °C)	
	Rm, MPa	Area of Rupture	Weld Face	Weld Root	HAZ	Weld
Joint no. 1	1053	HAZ	180	180	46	30
Joint no. 2	1068	HAZ	180	180	40	30

* Average result of two measurements; ** average result of three measurements, ^1^ standard deviation σ = 9.9.
